# Online Adaptive Radiotherapy for an Elderly Patient With Locally Advanced Pancreatic Cancer

**DOI:** 10.7759/cureus.71382

**Published:** 2024-10-13

**Authors:** Dazhen Jiang, Jin Peng, Hui Liu, Xiaoyong Wang, Fuxiang Zhou

**Affiliations:** 1 Radiotherapy Center, Hubei Key Laboratory of Tumor Biological Behaviors, Hubei Cancer Clinical Study Center, Zhongnan Hospital of Wuhan University, Wuhan, CHN; 2 Department of Radiation and Medical Oncology, Hubei Key Laboratory of Tumor Biological Behaviors, Hubei Cancer Clinical Study Center, Zhongnan Hospital of Wuhan University, Wuhan, CHN

**Keywords:** dosimetric comparison, fan-beam computed tomography, intensity-modulated radiation therapy, locally advanced pancreatic cancer, online adaptive radiotherapy

## Abstract

This case report details the treatment process and outcomes of an elderly patient with locally advanced pancreatic cancer (LAPC) managed with online adaptive radiotherapy (ART) guided by fan-beam computed tomography. The patient exhibited significant tumor regression (partial response) during the treatment course. Follow-up imaging one year after treatment confirmed sustained tumor remission and maintained a good quality of life. These findings highlight the potential efficacy of online ART in the management of LAPC.

## Introduction

The prognosis for patients with locally advanced pancreatic cancer (LAPC) remains dismal [1]. The current standard of care typically involves chemotherapy and radiotherapy [2]. However, for elderly patients or those deemed unsuitable for chemotherapy, radiotherapy alone becomes the primary treatment modality. Unfortunately, pancreatic tumors are frequently located near critical structures such as the duodenum and stomach, constraining the deliverable radiation dose to the tumor. This limitation often results in local failure or metastasis, leading to rapid tumor progression.

Stereotactic body radiation therapy (SBRT) has emerged as a promising technique due to its ability to deliver highly ablative radiation doses over a few fractions [3,4]. A study by Park et al. demonstrated improved quality of life and acceptable acute toxicity with a five-fraction SBRT regimen compared to intensity-modulated radiation therapy [5]. However, despite SBRT's increased accuracy and precision, escalating the prescribed dose without compromising safety remains challenging. The pancreas's sensitivity to interfraction motion during the gastrointestinal peristalsis and filling degree is a significant concern. Furthermore, adjacent organs at risk (OARs), including the stomach, duodenum, and small intestine, are highly sensitive to radiation, necessitating stringent dose limitations to prevent significant treatment-related toxicity [6].

Adaptive radiotherapy (ART) addresses the challenges of over-irradiating OARs and under-irradiating the planning target volume (PTV) due to daily anatomical variations. ART encompasses offline and online techniques, where the initial radiotherapy plan is adjusted based on changes in tumor size, morphology, and location to achieve "adaptive" irradiation for each fraction. Online ART involves scanning the patient's radiotherapy area before each session to capture real-time changes in the tumor and surrounding organs. The treatment plan is then adjusted to maximize tumor control while minimizing exposure to normal tissues.

We conducted a patient diagnosed with LAPC. The gross tumor volume (GTV) delineates the visible extent of the tumor on imaging, while the PTV was generated by expanding 5 mm from the GTV and ensuring a 3 mm distance from the small intestine, duodenum, and stomach simultaneously. Treatment planning was executed using the United Imaging Healthcare Treatment Planning System workstation (United Imaging HealthCare Co., LTD., Shanghai, China). The control group underwent evaluation based on daily validated fan-beam CT (FBCT) scans, assessing both the dose delivered to actual OARs and the target volume. Patients in the study group received daily validated FBCT-guided ART plans, with a focus on mean dose assessment of both the target volume and OARs. Subsequently, we also compared the average outcomes of each treatment fraction between IGRT and online ART.

This report provides a comprehensive account of the treatment process and outcomes of a pancreatic cancer case managed with online ART guided by FBCT, highlighting the potential benefits of this approach.

## Case presentation

**Figure 1:**
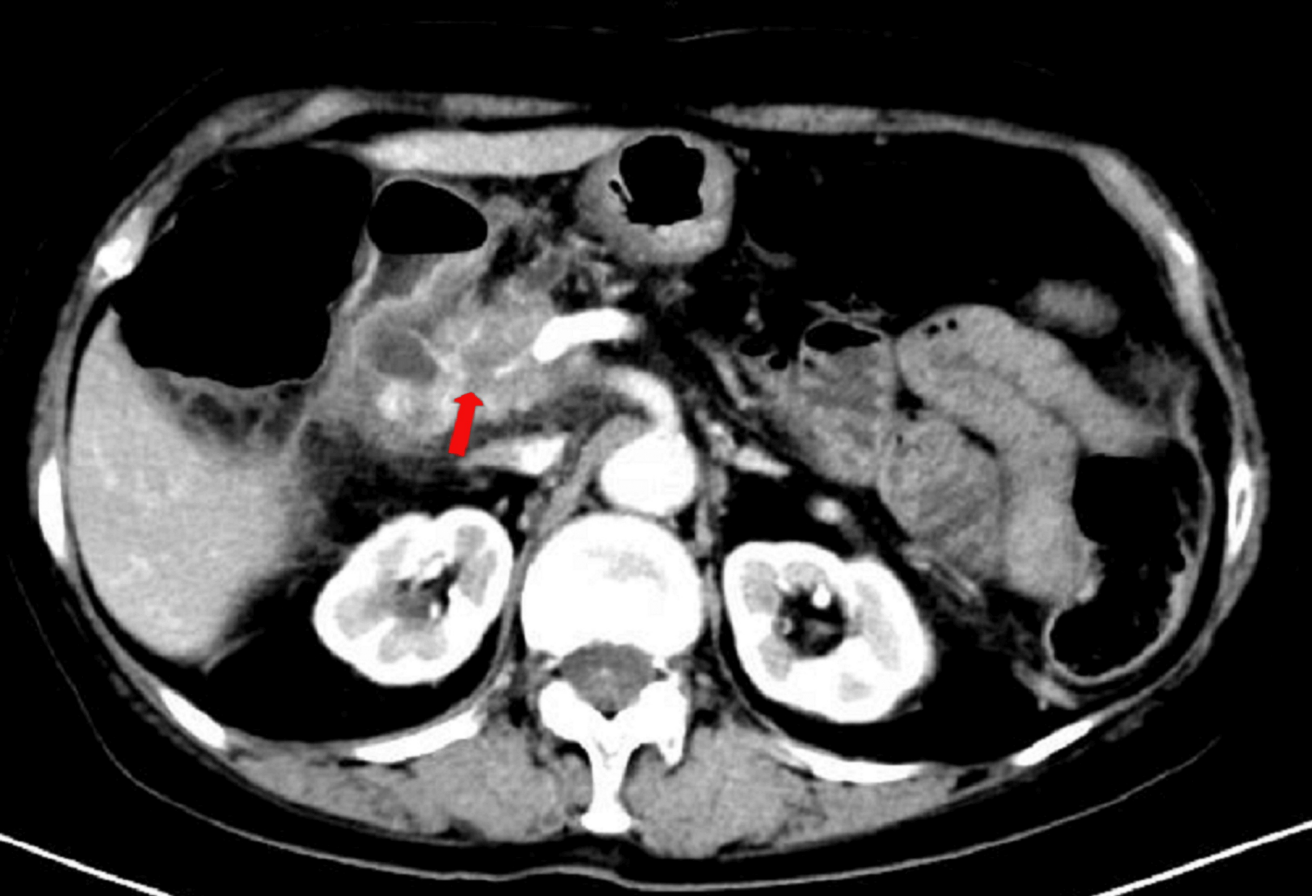
Enhanced CT on May 28, 2023, showing a soft tissue shadow in the hepatoportal-pancreatic head region with dilatation of the intrahepatic bile ducts, common bile duct, and pancreatic duct, and tumor invasion encircling the common hepatic artery and portal vein (red arrow) CT: computed tomography

A 77-year-old woman was admitted to the hospital on May 29, 2023, for evaluation of a pancreatic mass detected during a physical examination over one month before. The patient exhibited no significant abdominal pain, bloating, or discomfort. An abdominal computed tomography (CT) (Figure 1) showed a soft tissue shadow in the hepatoportal-pancreatic head area, with dilatation of the intrahepatic bile ducts, bile ducts, and pancreatic ducts. Tumor invasion and spongy degeneration of the portal vein were also observed.

The patient's medical history included hypertension and coronary atherosclerosis for two years, a cholecystectomy 27 years ago, and endoscopic retrograde cholangiopancreatography dilatation for bile duct stenosis 20 years ago. She also had a history of cervical spondylosis for more than 10 years and lumbar disc herniation for eight years. Physical examination revealed emaciation, no yellowing of the skin and sclera, and a performance score of 1. No palpable lymph nodes were enlarged, and respiratory sounds were clear.

**Figure 2:**
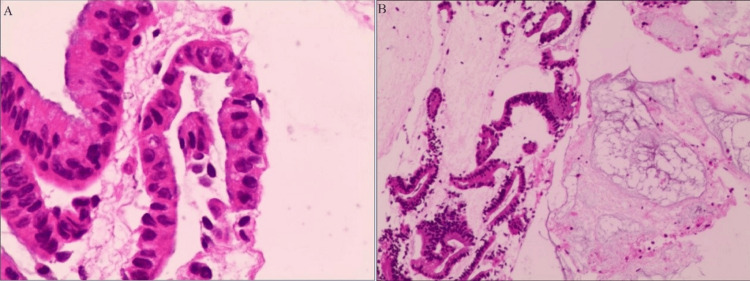
Ultrasound gastroscopy-guided biopsy of the pancreatic head mass showing adenocarcinoma. (A) 100×. (B) 400×

The patient was admitted to the hospital with an elevated cancer antigen 19-9 (CA19-9) level of 456.00 U/mL. Routine blood tests and liver and kidney function assessments were generally within normal limits. Ultrasound gastroscopy with puncture biopsy of the pancreatic head mass revealed adenocarcinoma. Immunohistochemistry showed human epidermal growth factor receptor 2 (0, negative), Claudin18.2 (0, negative), P53 (wild type), Ki-67 (proliferation index about 60%), MLH1(+), PMS2(+), MSH2(+), MSH6(+), and programmed death-ligand 1 (combined positive score 0). In addition, the Epstein-Barr encoding region (in situ hybridization) was negative. Pancreatic head cancer (cT4N0M0) was diagnosed, as shown in Figure 2.

Treatment program

The patient, presenting in poor general condition and with limited tolerance for chemotherapy, declined intravenous chemotherapy and commenced radiotherapy in June 2023 for a mass in the hepatoportal-pancreatic head region. Online ART was employed as the primary treatment modality. During the radiotherapy course, the patient received synchronous tegafur-uracil (40 mg, twice daily, Monday through Friday). Following radiotherapy, oral tegafur-uracil maintenance therapy was administered for five cycles but was discontinued due to the development of grade III thrombocytopenia and dry eye.

Treatment planning and dose prescription

The GTV was delineated based on imaging, and the PTV was generated by expanding 5 mm from the GTV, sparing the small intestine, duodenum, and stomach by 3 mm. The prescribed dose for the PTV-GTV was 45 Gy delivered in five fractions (45Gy/5F). The plan ensured that at least 100% of the prescription dose covered 95% of the target volume. OARs included the duodenum, stomach, adjacent small intestine, kidneys, liver, and spinal cord, with a dose limit of 39 Gy for the duodenum and small intestine in volumes ≤2 cc.

Online ART plan

**Figure 3:**
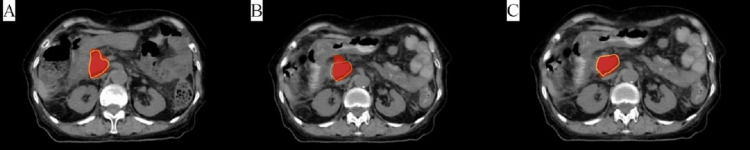
Dose distribution for 45Gy, showing target volume coverage of different plans. (A) Dose distribution of the initial plan. (B) Recalculation of the initial plan on FBCT images during online ART. (C) Adaptive plan FBCT: fan-beam computed tomography; ART: adaptive radiotherapy

All patients underwent radiation treatment planning at the United Imaging Kilovoltage Computed Tomography-Linac workstation (Shanghai, China), and the plan was designed by the united radiation therapy (uRT) treatment planning system (United Imaging HealthCare Co., LTD.). The ART plan was developed using a convolution dose calculation algorithm, and a nine-field coplanar static intensity-modulated radiation treatment plan with a 6-MV photon was delivered. The plan was optimized and assessed by oncologists and physicists using clinical goal sheets, dose-volume histograms, 3D dose distributions (Figure 3), and other assessment tools. The treatment beam was scheduled upon acceptance, and the plan was transferred to the treatment delivery administrator.

Prior to each treatment fraction, FBCT was acquired and rigorously matched to the planned CT to ascertain the position of the patient and the tumor. Quality assurance of the ART plan was ensured using the uRT system, providing an online electronic portal imaging device-based in vivo dosimetry solution as an additional monitoring tool during treatment.

Dosimetric comparison

**Figure 4:**
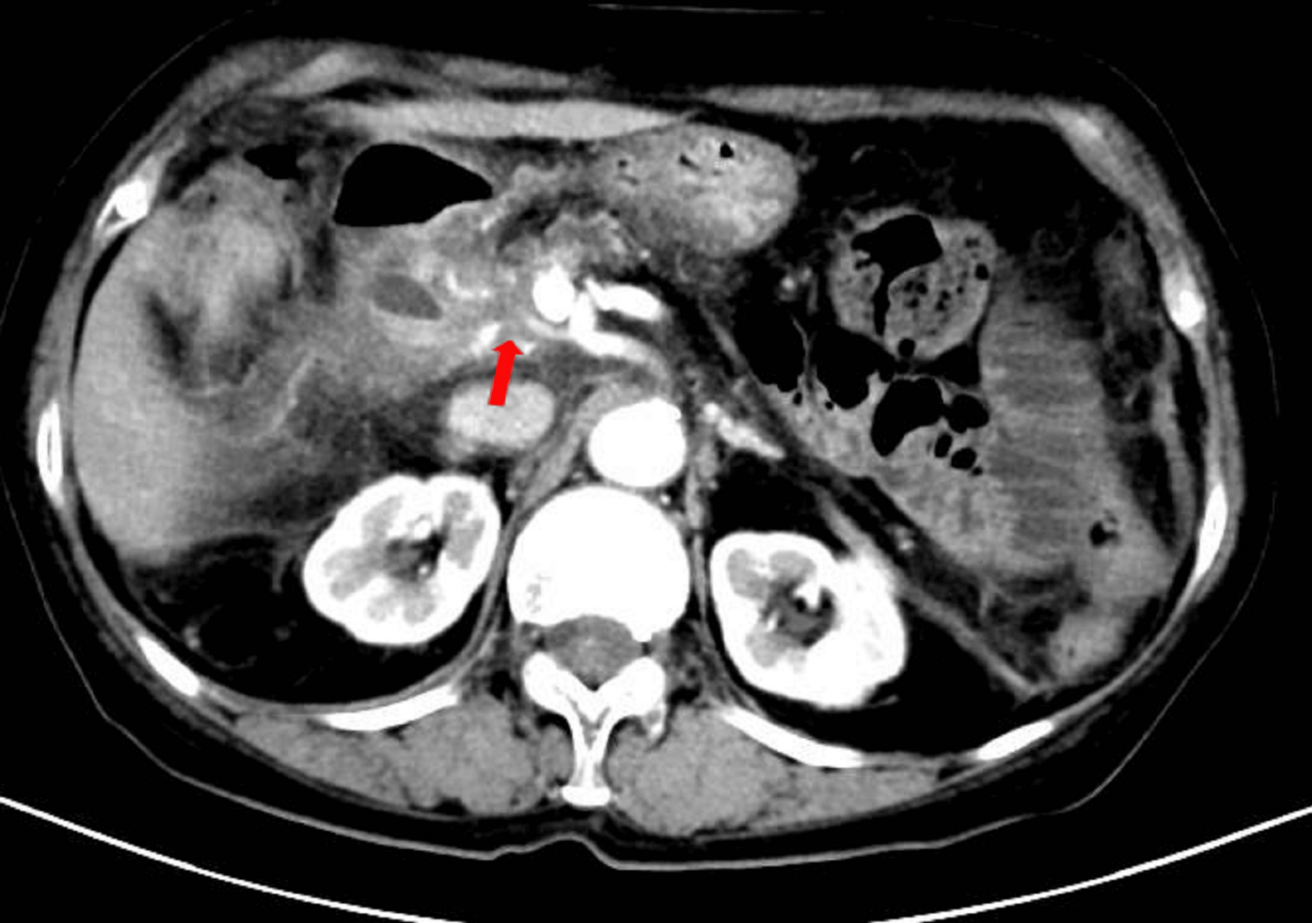
Enhanced CT on August 26, 2023, showing more significant oozing next to the pancreatic head mass and increased pelvic fluid, with no significant changes elsewhere. The tumor response has been assessed as SD (red arrow) CT: computed tomography; SD: stable disease

**Figure 5:**
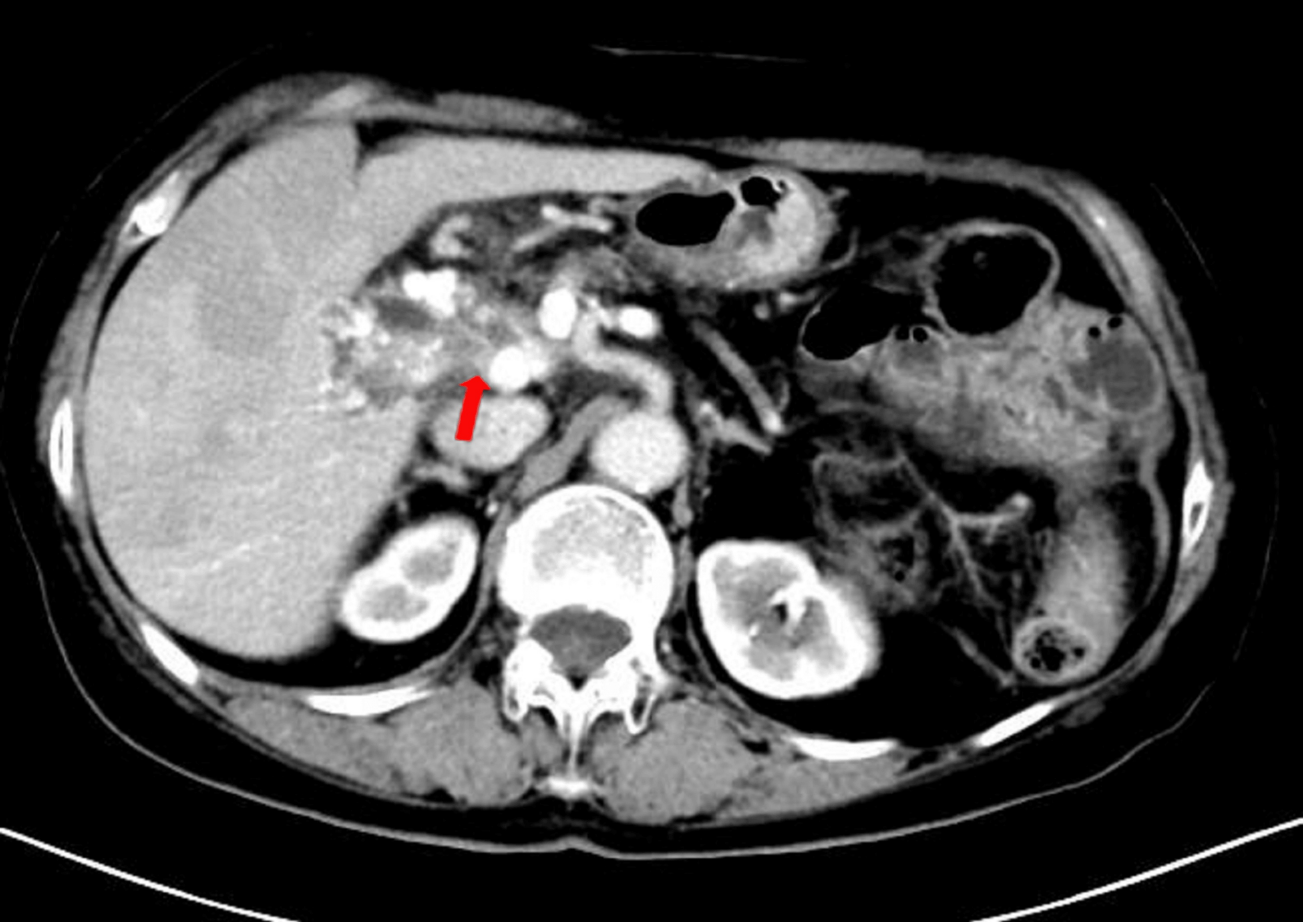
Follow-up CT in January 2024, showing continued reduction in pancreatic head tumor size, assessed as PR (red arrow) CT: computed tomography; PR: partial response

**Figure 6:**
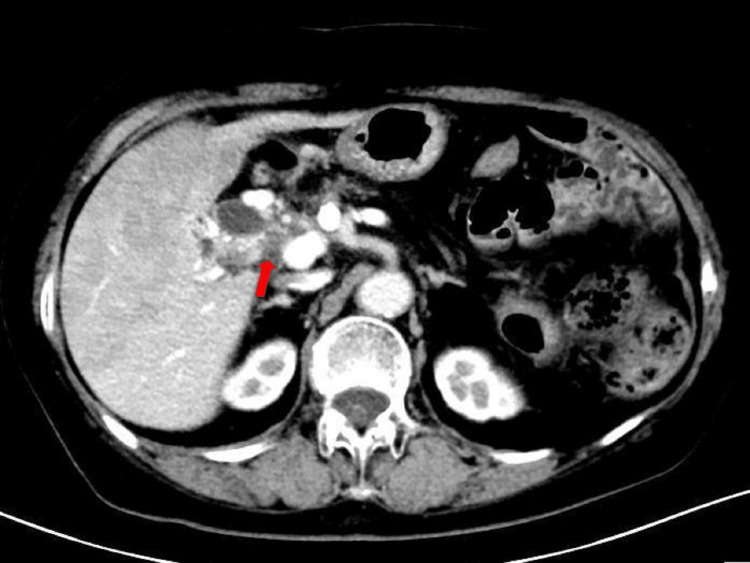
Follow-up CT in July 2024, showing that the tumor continues to decrease (red arrow) CT: computed tomography

**Figure 7:**
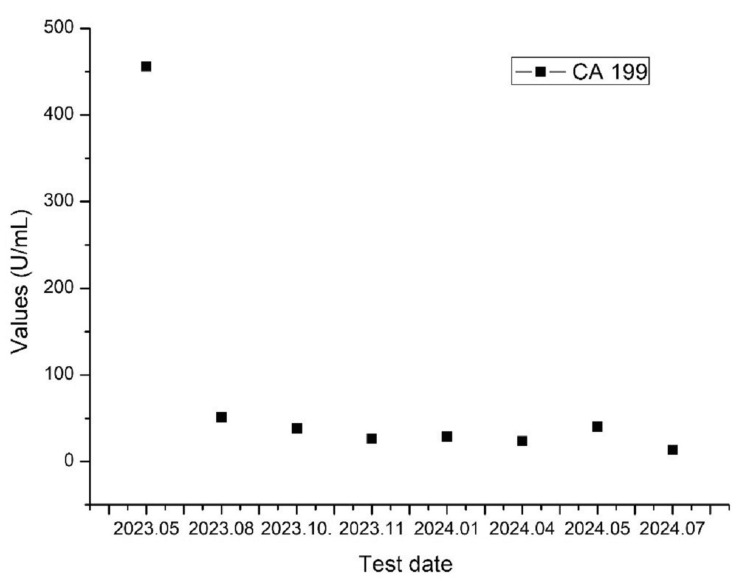
CA19-9 test values, showing that the value of CA19-9 is continuously decreasing CA19-9: cancer antigen 19-9

The patient experienced no significant adverse effects during treatment and tolerated it well. Grade III thrombocytopenia occurred after radiotherapy but recovered after platelet-boosting therapy. As shown in Figure 4, six months after treatment, the patient's tumor volume had decreased by 50%, with a partial response (PR) observed. One year later, CT (Figures 5, 6) and MRI indicated sustained PR, and the patient maintained a good quality of life. As shown in Figure 7, the value of CA19-9 was continuously decreasing.

## Discussion

This case report illustrates the promising potential of online adaptive radiotherapy (OART) for managing LAPC, particularly in patients who are not candidates for chemotherapy. The patient's sustained tumor regression and maintenance of a good quality of life underscore the efficacy and safety of OART. Future research should focus on addressing existing technical challenges and optimizing ART protocols to improve treatment outcomes for pancreatic cancer patients.

The findings from this case highlight the potential benefits of integrating OART into pancreatic SBRT. However, several technical challenges must be overcome before ART can be widely adopted in clinical practice. A significant concern is the duration patients spend on the treatment table during ART. Despite graphics processing unit (GPU) acceleration facilitating rapid processing, targeted reoptimization, and image warping, other procedural steps remain time-consuming. Specifically, tasks such as evaluating deformation delineation, redetermining the PTV, and planning for reoptimization require substantial time investment. Additionally, the ART process necessitates rigorous independent quality assurance prior to treatment delivery. Addressing these challenges through further technological refinement and optimization is essential for the effective integration of ART into routine clinical workflows.

In this study, FBCT was employed, offering superior image quality compared to conventional megavoltage computed tomography and cone-beam computed tomography (CBCT) [7,8]. The diagnostic-grade FBCT utilized for simulation proved to be significantly more time-efficient than CBCT-based ART and effectively addressed issues such as deformation correction and Hounsfield unit value adjustments of CT images [8-10]. However, challenges persisted in imaging pancreatic tumors, which proved difficult to delineate even with intravenous contrast in high-resolution pancreatic protocol CT scans. In this study, patients were instructed to ingest 100 mL of water containing 5 mL of contrast agent, split into two doses: one 15 minutes before treatment and one immediately before. While this method facilitated the identification of the small intestine, duodenum, and stomach, it did not permit precise tumor contouring, complicating the verification of contour distortion and metastasis. Additionally, respiratory motion was not accounted for in this study. Future research should incorporate respiratory gating into ART, necessitating the use of 4D FBCT and advanced image processing techniques.

This case demonstrates that, in the absence of chemotherapy, pancreatic radiotherapy alone achieved over one year of tumor control with an excellent quality of life. This suggests that online ART alone may serve as an effective stand-alone treatment for certain pancreatic cancer patients, particularly those ineligible for chemotherapy.

Furthermore, after treatment, the patient experienced significant improvements in pain reduction, physical function recovery, and psychological well-being. These findings suggest that OART not only effectively controls tumors but also alleviates the physical and psychological burdens associated with surgery and chemotherapy, thereby enhancing overall patient quality of life.

## Conclusions

This case report indicates that for certain patients with LAPC who are unsuitable for chemotherapy, radiotherapy alone can provide prolonged tumor control while maintaining a good quality of life. Although this is a single case report, it suggests that OART may emerge as a significant treatment modality for LAPC. Further clinical studies and the accumulation of additional cases are necessary to substantiate its safety and effectiveness.
